# Molecular dynamics simulations of displacement cascades in LiAlO_2_ and LiAl_5_O_8_ ceramics

**DOI:** 10.1038/s41598-024-51222-4

**Published:** 2024-01-22

**Authors:** Ankit Roy, Andrew M. Casella, David J. Senor, Weilin Jiang, Ram Devanathan

**Affiliations:** Pacific Northwest National Laboratory, Richland, WA 99354 USA

**Keywords:** Nuclear fusion and fission, Computational methods

## Abstract

Molecular dynamics was employed to investigate the radiation damage due to collision cascades in LiAlO_2_ and LiAl_5_O_8_, the latter being a secondary phase formed in the former during irradiation. Atomic displacement cascades were simulated by initiating primary knock-on atoms (PKA) with energy values = 5, 10 and 15 keV and the damage was quantified by the number of Frenkel pairs formed for each species: Li, Al and O. The primary challenges of modeling an ionic system with and without a core–shell model for oxygen atoms were addressed and new findings on the radiation resistance of these ceramics are presented. The working of a variable timestep function and the kinetics in the background of the simulations have been elaborated to highlight the novelty of the simulation approach. More importantly, the key results indicated that LiAlO_2_ experiences much more radiation damage than LiAl_5_O_8_, where the number of Li Frenkel pairs in LiAlO_2_ was 3–5 times higher than in LiAl_5_O_8_ while the number of Frenkel pairs for Al and O in LiAlO_2_ are ~ 2 times higher than in LiAl_5_O_8_. The primary reason is high displacement threshold energies (E_d_) in LiAl_5_O_8_ for Li cations. The greater E_d_ for Li imparts higher resistance to damage during the collision cascade and thus inhibits amorphization in LiAl_5_O_8_. The presented results suggest that LiAl_5_O_8_ is likely to maintain structural integrity better than LiAlO_2_ in the irradiation conditions studied in this work.

## Introduction

Gamma lithium aluminate (γ-LiAlO_2_) is used in the production of tritium for the strategic stockpile and is being considered for fusion reactor blanket applications. It has excellent thermomechanical durability and low volumetric swelling when exposed to neutron irradiation^1^. It is used in tritium-producing burnable absorber rods (TPBARs)^2^ in the form of high density pellets enriched in ^6^Li. In the first irradiation study of γ-LiAlO_2_^3^ under 200 keV electrons at room temperature, the formation of a secondary LiAl_5_O_8_ phase was noticed due to precipitation. This precipitation was attributed to the Li and O atomic displacements induced by irradiation, given by the reaction, 5 LiAlO_2_ → LiAl_5_O_8_ + 4 Li (displaced from lattice site) + 2 O (displaced from lattice site), which was also confirmed by a recent report^4^. Since the displacement of Li from the lattice can lead to potential degradation of TPBAR performance, we simulated atomic displacement of Li in cascades in these two ceramics via molecular dynamics (MD) to understand their radiation response.

Radiation damage is a consequence of the radiation transport in a solid material where atomic displacements, radiation induced segregation, radiation induced phase transformation and radiation induced microstructure evolution are a few major types of damages^5^. Most metallic alloys show a combination of these damages, for example, $\alpha +\beta$ dual phase Ti–6Al–4V alloys have been seen to develop an $\omega$ phase after irradiation (irradiation induced phase transformation) and also form defect clusters and dislocation loops as a result of atomic displacements^6–8^. On the other hand lithium ceramics have been reported to have undergone amorphization due to atomic displacements.^9,10^ This study focuses on where an atom gets knocked from its original site, leaving behind a vacancy. When a high energy neutron or a recoiling atom collides with atoms in the lattice, a damage cascade is initiated with point defects leading to defect clusters that can cause degradation of material properties. In Li-containing ceramics, the accumulation of point defects can cause amorphization of the crystal lattice^11,12^ and affect the tritium release^13–15^.

With regards to γ-LiAlO_2_, Tsuchihira et al.^16^ performed cascade simulations and found that Li forms the largest number of defects. They complemented this observation by calculating the threshold displacement energies for Li, O and Al. Li defects are predominant due to the low displacement energy of 22 eV for Li in comparison to 84 eV for aluminum and 37 eV for oxygen. A recent MD study investigated irradiation damage in γ-LiAlO_2_ and found that Li defects account for about 70% of the damage and LiO_4_ tetrahedra are more susceptible to damage than the AlO_4_ tetrahedra^17^. Although there has been computational and experimental research into the radiation response of γ-LiAlO_2_, much less is known about these phenomena in LiAl_5_O_8_. It is known that during irradiation, due to the rapid transmutation of Li into tritium (T) and helium, the Li in γ-LiAlO_2_ is rapidly consumed which leads to the formation of considerable amount of the secondary phase LiAl_5_O_8_^18^.

**Figure 1:**
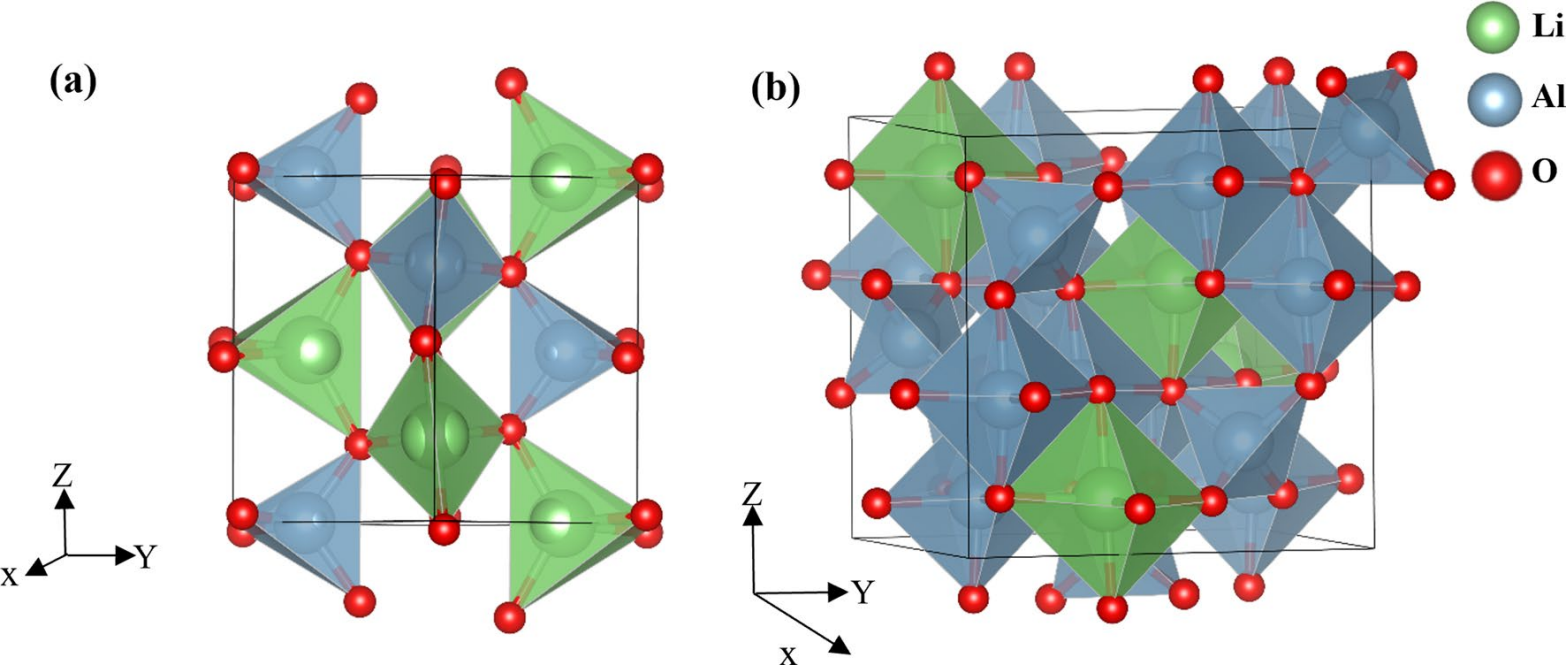
The crystal structure of γ-LiAlO_2_ showing (**a**) the interconnected tetrahedra of Li/Al type (green/blue). (**b**) The crystal structure of LiAl_5_O_8_ showing the interconnected octahedra of Li/Al type (green/blue).

An understanding of the structures of the two ceramics is essential before investigating the structural damage caused due to irradiation. The γ-LiAlO_2_ crystal is comprised of a network of distorted XO_4_ tetrahedra (X = Li, Al) arranged in three dimensions. Figure 1a shows each tetrahedron has a Li/Al metal atom at the center and oxygen atoms at the corners. There is one common edge for every LiO_4_-AlO_4_ tetrahedron. This arrangement forms quadrangular channels along the [001] direction thus generating a linear order of octahedral voids^19^.

The LiAl_5_O_8_ crystal has a network of Li/Al–O_6_ octahedra linked by Al–O_4_ tetrahedra as depicted in Fig. 1b. It contains 4 Li, 20 Al and 32 O atoms in a unit cell. The stoichiometry can be expressed as (Al)_tet_(Li_1/2_Al_3/2_)_oct_O_4_ where every Li ion is centered in an octahedron at 4d Wyckoff sites, 3 out of 5 Al ions are contained in octahedra at 12d Wyckoff sites while the other 2 Al are in tetrahedra at 8c Wyckoff sites.

There is a paucity of information, beyond the crystal structure, about LiAl_5_O_8_ in the literature. The present work aims to address this gap by performing displacement cascade simulations in these two ceramics using MD and comparing the tolerance to radiation and amorphization in the two ceramics. The cascade simulations were complemented with calculations of the displacement threshold energy (E_d_), which is the minimum energy needed to displace any given atom from its original site, leaving behind a permanent defect. The results highlight the differences in defect production in the two ceramics that could be crucial in interpreting the susceptibility of these ceramics to radiation-induced amorphization.

## Computer simulation methods

### Potential selection

With regards to MD potentials for the LiAlO_2_ system, Jacob et al. developed a potential in 1996^20^ and Tsuchihira–Oda–Tanaka^21^ developed another potential in 2009. Jacob’s potential did not generate a stable structure^17^ and was not considered for our work. The TOT potential has been well established for the LiAlO_2_ system but the charges in the TOT potential are 0.7 for Li, − 1.1 for O and 1.5 for Al, which is not transferrable to the LiAl_5_O_8_ system because the charges would not add up to zero. For a fair comparison of displacement energetics in the two ceramics, we required a set of Li–Al–O interactions that can be used for both the LiAlO_2_ and LiAl_5_O_8_ systems. Therefore, we employed the potential proposed by Kuganathan et al.^22^ which maintains charge neutrality for both systems. We have implemented this potential and shown its accuracy for both systems in our previous work^9^ where we found that it reproduces bond lengths, melting points and densities of both ceramics with reasonable accuracy. This potential encompasses interactions of short range (i.e., van der Waals attraction and electron–electron repulsion) and long range (Coulombic). The short-range interactions were modeled using Buckingham potentials with the parameters listed in Table 1.

**Table 1:** Parameters of the Buckingham potential used in the MD simulations of Li-Al-O systems taken from^23–25^.

Two-body [${\varnothing }_{ij}\left({r}_{ij}\right)={A}_{ij}{\text{exp}}\left(-\frac{{r}_{ij}}{{\rho }_{ij}}\right)-{C}_{ij}/{r}_{ij}^{6}$]					
Interaction	A (eV)	$\rho$ (Å)	C (eV. Å^6^)	Y (e^−^)	K (eV. Å^−2^)
Al^3+_^O^2−^	1109.92381	0.31540	0.00	3.00	99,999
Li^+_^O^2−^	632.1018	0.2906	0.00	1.00	99,999
O^2−_^O^2−^	12,420.5	0.2215	29.07	− 2.80	31.0

The combined expression of Buckingham and Coulombic potential is given by

$$E={A}_{ij}{\text{exp}}\left(-\frac{{r}_{ij}}{{\rho }_{ij}}\right)-\frac{{C}_{ij}}{{r}_{ij}^{6}}+\frac{C{q}_{i}{q}_{j}}{\epsilon r}$$

where C is an energy conversion constant, q_i_ and q_j_ are charges on the two atoms, $\epsilon$ is the dielectric constant, ${\rho }_{ij}$ is length parameter that depends on the ionic pair under consideration.

**Figure 2:**
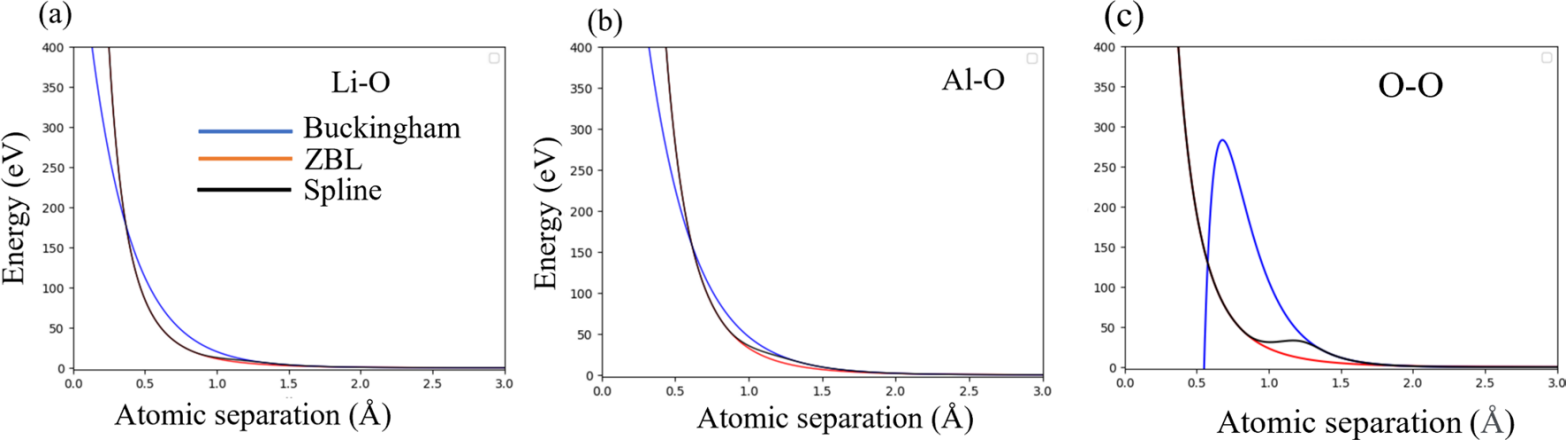
Splined Buckingham/ZBL potential for Li–O, Al-O and O–O interactions.

When the atoms were within a distance of 0.5 Å, the Buckingham potential was smoothly combined with the ZBL^26^ potential to give a combined effective short range interaction. The Buckingham potentials were splined with ZBL using the atsim.potentials python code^27^ as shown in Fig. 2a–c for Li–O, Al–O and O–O interactions. The Fig. 2c shows the error in the Buckingham potential (blue line) which dips to a negative value below 0.6 Å. While the ZBL interaction is more accurate in this region, it is not very accurate in distances > 0.6 Å. Thus splining of the two potential creates a more accurate resultant potential at all distance below and above the 0.6 Å distance. The cation self interactions and the Li-Al interactions were assumed to have only ZBL interactions at short distances and Coulombic interactions at larger distances just like the Ca–Ca in refs.^28^.

We have performed an extensive validation of the potentials in our previous manuscript^9^ where we validated the bond distances and melting points for both ceramics with corresponding experimental and DFT data with reasonable accuracy. We also compared the diffusion coefficients (Li^+^ diffusion in LiAl_5_O_8_, D_0_ = 3.17 × 10^−11^ m^2^/s, Li^+^ diffusion in LiAlO_2_, D_0_ = 4.02 × 10^−10^ m^2^/s at 600 K) and migration barriers obtained by these potentials to the previously reported values and found a close agreement. For a preliminary validation in the current work, we have rechecked the reproducibility of density for both ceramics. For the density calculations, the systems were first energy minimized by the conjugate gradient method with an energy tolerance of 10^−30^ eV and a force tolerance of 10^−30^ eV/Å. Then the systems were initiated at 300 K under the isothermal-isobaric (NPT) ensemble and equilibrated for 100 ps after which the density values are calculated in LAMMPS, as done in previous works^29,30^. The density values obtained are 2.48 gm/cc for LiAlO_2_ and 3.42 gm/cc for LiAl_5_O_8_ which are well in agreement with 2.64 gm/cc and 3.64 gm/cc, respectively reported in The Materials Project database^31^. Therefore the validity of these potentials have been sufficiently established for the present simulations. Other parameters like lattice constants, bond distances and melting points have been validated in details in our previous work^9^ that highlights the capability of this potential to be used with fair accuracy for both ceramics.

Two separate hypothetical particles (one as core, another one as shell, with charges of + 0.8 and − 2.8, respectively) were used for modeling O ions in our simulations. The core shell model is a method to simulate ionic systems by introducing polarizability in the system^32^. The core and shell model was constructed for O atoms using the ATOMSK toolkit^33^. The interaction between the core and shell of each oxygen atom is expressed by a spring force as

1 $${U}_{c-s}=k{r}_{c-s}^{2}$$

where r_c-s_ designates the separation distance between the core and shell and *k* is the parameterized spring constant taken to be as 31 eV/Å^2^^22^. These potentials have been previously used and validated and have been found to produce a stable structure for LiAlO_2_^22^. The Frenkel defect energy for Li was found to be 1.44 eV using these potential parameters^22^.

As an investigative effort we also model the system without the core–shell model for oxygen to draw a comparison between the two methods (with and without O shells). While modeling the system without the O core–shell, we combined the attributes of both core and shell and assign them collectively to each individual O particle in the system.

### Atomic displacement cascade simulations

We used the LAMMPS^34^ code for atomistic simulations of displacement cascades on 21 × 21 × 21 unit cells along the x, y and z directions with periodic boundaries on all sides. The supercell size was around 11 nm × 11 nm × 13 nm containing about 225,000 atoms. Thermal equilibration was carried out in the NPT ensemble with 1 fs timesteps for 5 ps. Cascades were simulated keeping the number of particles fixed in the isolated system and at a constant volume (NVE ensemble). The size of the supercell was chosen to contain the displacement cascade within the boundaries. However, it is challenging to perform displacement cascade simulations on systems that involve core–shell interactions due to the concern that the shell center may drift away from the core center due to excessive kinetic energies during PKA initiation, and thus the core–shell separation may lead to simulation failure. To address this challenge, we adopted a variable timestep approach that calculates the timestep size (dt) such that the fastest particle in the whole system was allowed to travel only a certain designated maximum distance of X_max_ = 0.003 nm per timestep. This approach reset the dt in every step so that no atom in the system was displaced more than the specified X_max_ based on the atom forces and velocities. Additionally, all simulations were carried out with a 5 Å thick Langevin bath maintained at 300 K to dissipate the heat generated by the high energy PKA. This thermostat had a significant role in limiting the maximum temperature rise during PKA initiation but had a negligible effect on the defect production due to the small fraction of atoms included in its volume^35^. The velocities of the atoms in the thermostat region were rescaled to the corresponding value at 300 K at every step so that this thermostat simulated the dissipation of heat energy from the cascade region into the bulk. A Li atom at the center of the box was chosen as the PKA and was initiated in 8 randomly chosen directions as shown in Table 2 with velocities corresponding to kinetic energies of 5, 10 or 15 keV. For the systems without the oxygen shells, we only simulated 5 directions (A, B, C, D and E) of the PKA and only at 15 keV each just to draw a comparison between the performances of the forcefield with and without the oxygen shells.

**Table 2:** Li PKA initiation directions for all three PKA energies (5, 10 and 15 keV).

Direction	$\widehat{i}$	$\widehat{j}$	$\widehat{k}$
A	0.2	0.13	0.97
B	0.63	0.6	0.49
C	0.7	0.69	0.19
D	0.3	0.94	0.18
E	0.96	0.23	0.16
F	0.43	0.44	0.79
G	0.8	0.6	0.11
H	0.81	0.49	0.31

The main principle for deciding the simulation cell size is to prevent the displacement cascade from overlapping with itself due to its expansion beyond the periodic cell boundaries. This condition is generally governed by the PKA energy^36^. If it is satisfied, the cell size can be considered appropriate for the simulation. For example ref.^36^ used only 140,608 (26 × 26 × 26 unit cells) for a 20 keV PKA simulation in indium arsenide and did not observe any overlap of the cascade with itself. Beland et al.^37^ used a simulation cell with 256,000 atoms for Ni, NiFe and NiCo alloys for 1 and 10 keV PKA simulations but used a larger simulation cell only for 20 to 40 keV simulations. Another work^38^ used a 18.5 × 18.5 × 18.5 nm cell for studying 15 and 20 keV PKA cascades in FeCr systems which is a comparable size to the simulation cell used in the present work for 15 keV. Even though we used a small cell, we investigated the cascade evolution for all directions in the highest PKA energy of our work i.e. 15 keV.

In order to ensure that the size of the chosen simulation box is optimum such that there are no variations in the results of defects produced due to the box size, we performed one simulation in a larger 23 nm × 23 nm × 27 nm cell containing about 1,800,000 (1.8 million) atoms (8 times that in the smaller simulation cell) and compared the results to the smaller 11 nm × 11 nm × 13 nm cell, containing 225,000 atoms. A comparison of the number of Frenkel pairs produced as a result of displacement cascades was performed for both these cells, by launching a 15 keV PKA in both the small and big simulation cells in an identical direction. All factors remaining identical, except the size of the simulation box, we compare the number of Frenkel pairs generated and the cascade evolution in both the cells.

All visualizations have been performed using Ovito^39^. Defects were identified using the Wigner Seitz^40^ algorithm inbuilt in Ovito. The Wigner–Seitz (WS) method operates by defining a spatial partitioning scheme using Voronoi cells around each atomic site. Mathematically, a Voronoi cell (or a Wigner–Seitz cell) around an atomic site is defined as the volumetric locus of points in space that are closer to that site than to any other site. Vacancies are identified as sites that have no atom associated with them. These sites, often referred to as “vacancy sites,” are those within the material where no atom is present. This detection is based on the concept of occupancy numbers associated with each WS cell. Any site with an occupancy number of zero indicates the absence of an atom, thus signifying a vacancy site. One limitation is that the method might not effectively capture vacancies that occur near the border or edge of the Voronoi cells. This could lead to an underestimation of vacancies, particularly in cases where atoms are located near the boundary of a Voronoi cell without being associated with a specific lattice site. To cope with this limitation multiple methods for visualization of cascades have been adopted to make sure the captured spread of the cascade is reasonably accurate.

**Figure 3:**
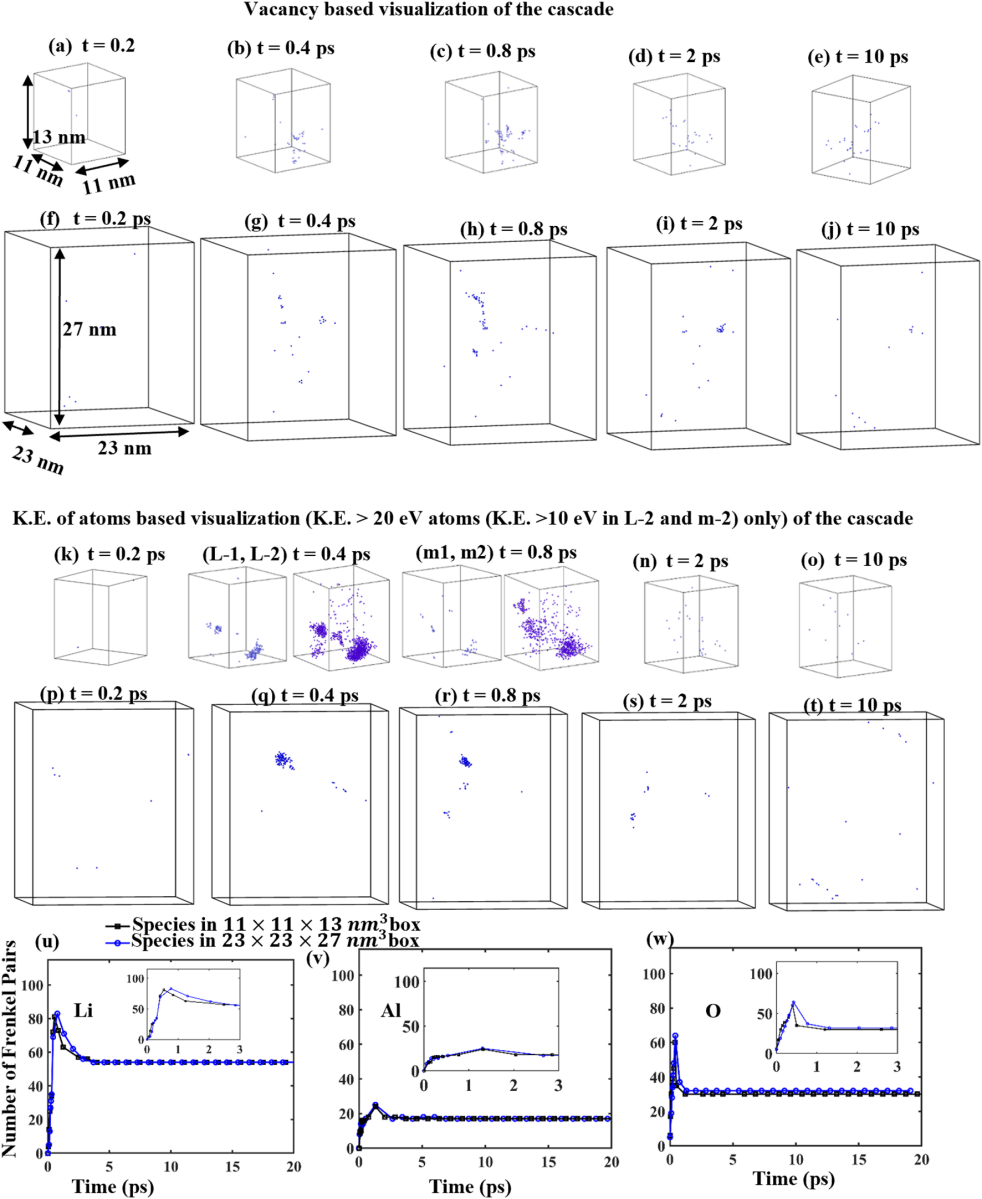
Visualization of the cascade evolution as a function of time for a 15 keV PKA based on vacancy cluster formation (**a**–**e**) for smaller simulation box and (**f**–**j**) for larger simulation box. Visualization based on energy (> 20 eV) of atoms (**k**–**o**) for smaller simulation box (L-2 and m2 show the atoms with energy > 10 eV) and (**p**–**t**) for larger simulation box. (**u**–**w**) Comparison of number of Frenkel pairs formed by a 15 keV Li pka in identical directions in the smaller and the larger simulation box. Inset in (**u**–**w**) shows a magnification of the 0–3 ps time frames.

There are two most common methods of visualizing the collision cascades, one based on the energies (or temperature) of the atoms and the other based on the point defect clusters formed. In the energy based visualization, atoms with higher energies (or temperatures) represent those within the cascade region^41^ and in the vacancy based visualization, the cascade is represented by the cluster of point defects (usually vacancies) that have been formed as a result of massive displacement of atoms from their lattice during their interaction with the cascade^42,43^. The present work visualizes the cascades according to both the energy based and the point defects (vacancy) based criterion. The vacancy cluster-based visualization is shown in Fig. 3a–e for the smaller box and Fig. 3f–j for the larger box, as a function of time. The peak of cluster formation is noticed at about 0.8 ps in both cases, when the cascade occupies the maximum volume in the box. The energy-based visualization is shown in Fig. 3k–o for the smaller box and Fig. 3p–t for the larger box, as a function of time. The atoms in blue represent all atoms in system with an energy higher than 20 eV. This value of 20 eV was chosen based on the displacement threshold energies of the Li, Al and O species as it is seen later in the paper, that some Li atoms may get displaced if imparted an energy of 20 eV or higher. From the energy-based visualization, a peak is noticed at around 0.4 ps Fig. 3l and q. Note that the peak in energy is noticed earlier than the peak in vacancy cluster formation because a high energy collision precedes the formation of a subsequent defect. From these visualizations it is clear that the cascades at the peak of the damage are well contained within the bounds of the smaller simulation box and its size does not show any significant variation in the larger box.

Generally, defects, such as vacancies or interstitials, are produced when the energy imparted to an atom exceeds the displacement threshold energy (E_d_). At energies below E_d_, individual atoms typically don't acquire enough energy to break away from their lattice positions, thus reducing the likelihood of defect generation. However, during the thermal spike phase in cascade simulations, it is possible to have energetic atoms and movement of a large number of atoms. These movements might cause transient local disorder or structural changes, but they might not generate the higher-energy residual defects associated with displacing atoms from their lattice sites. While the average displacement threshold energy for Li in LiAlO_2_ is approximately 35 eV, our chosen cutoff of 20 eV was based on the lowest E_d_ value observed among the 15 simulated directions. This criterion was already at the lower end of the cutoff range to ensure the identification of atoms significantly affected by the cascade events. However, by further reducing the cutoff to 10 eV as shown in Fig. 3 (L-2) and Fig. 3 (m2), we aim to incorporate atoms that might not meet the conventional defect criteria but contribute to local structural changes and disorder. The cascade now appears to be only slightly larger than when the cutoff is set to 20 eV but is well contained within the simulation box. This adjustment allows for a more comprehensive representation of the cascade dynamics, capturing a broader spectrum of atoms influenced by the cascade with energies closer to, yet below, the typical displacement threshold. Consequently, this refined criterion offers a more detailed and nuanced view of the cascade's impact on the material, shedding light on subtle but potentially critical alterations in the atomic structure during the cascade events.

A comparison of the number of Frenkel pairs generated in both big and small simulation boxes in Fig. 3u–w shows that almost an identical number of Frenkel pairs are generated for Li and Al but for O there is a total of 64 Frenkel pairs at the peak of the cascade in the larger box as compared to 60 in the smaller box. The surviving number of O vacancies is 31 in the larger box as compared to 30 in the smaller one. Since this variation will most certainly fall within the error margins in a large sample set, it can be safely assumed that the small and the large box in our study have an identical nature of displacement damage. Clearly, there is also no cascade overlap which justifies the choice of our smaller but optimum simulation box size.

The Wigner–Seitz analysis as built in OVITO^39^ was used to determine defects, specifically the number of Frenkel pairs in the system. Since the core and shell of oxygen atoms underwent relative displacements during the simulation, only the cores of oxygen atoms were considered, to avoid false identification of defects due to relative displacements of core–shell centers. Defects of each individual species were identified by ignoring the atoms of all other species and performing the Wigner–Seitz analysis on the atoms of one particular type under consideration. The total number of defects can be easily obtained by the summation of defects of the individual species. The specifics of the cascade simulation such as the variable timestep size change, temperature and K.E. variations are discussed in the results section.

### Threshold displacement energy calculations

Simulation cells contained around 4000 atoms with periodic boundaries, and were equilibrated for 5 ps at 0 K. Displacement simulations were performed using methods from Devanathan et al.^44^ where a certain kinetic energy is imparted to a selected atom in the simulation cell within the NVE ensemble. The atoms were initiated in 8 directions identical to those that were used in the cascade simulations so that the results can support the events observed in displacement cascades. Defects were identified 5 ps after the initiation of the selected atom. The kinetic energies were incremented by 1 eV starting from 15 eV, until a permanent defect was identified. The final E_d_ is the kinetic energy that produces a stable defect but has no defect production at [E_d_-1] eV. The E_d_ was only calculated for Al and Li atoms, as the O atoms were modelled as two separate particles (core and shell). Therefore, imparting a high KE to any O atom, posed a risk of core–shell separation during the supersonic and transonic phase of the cascade.

## Results and discussions

**Figure 4:**
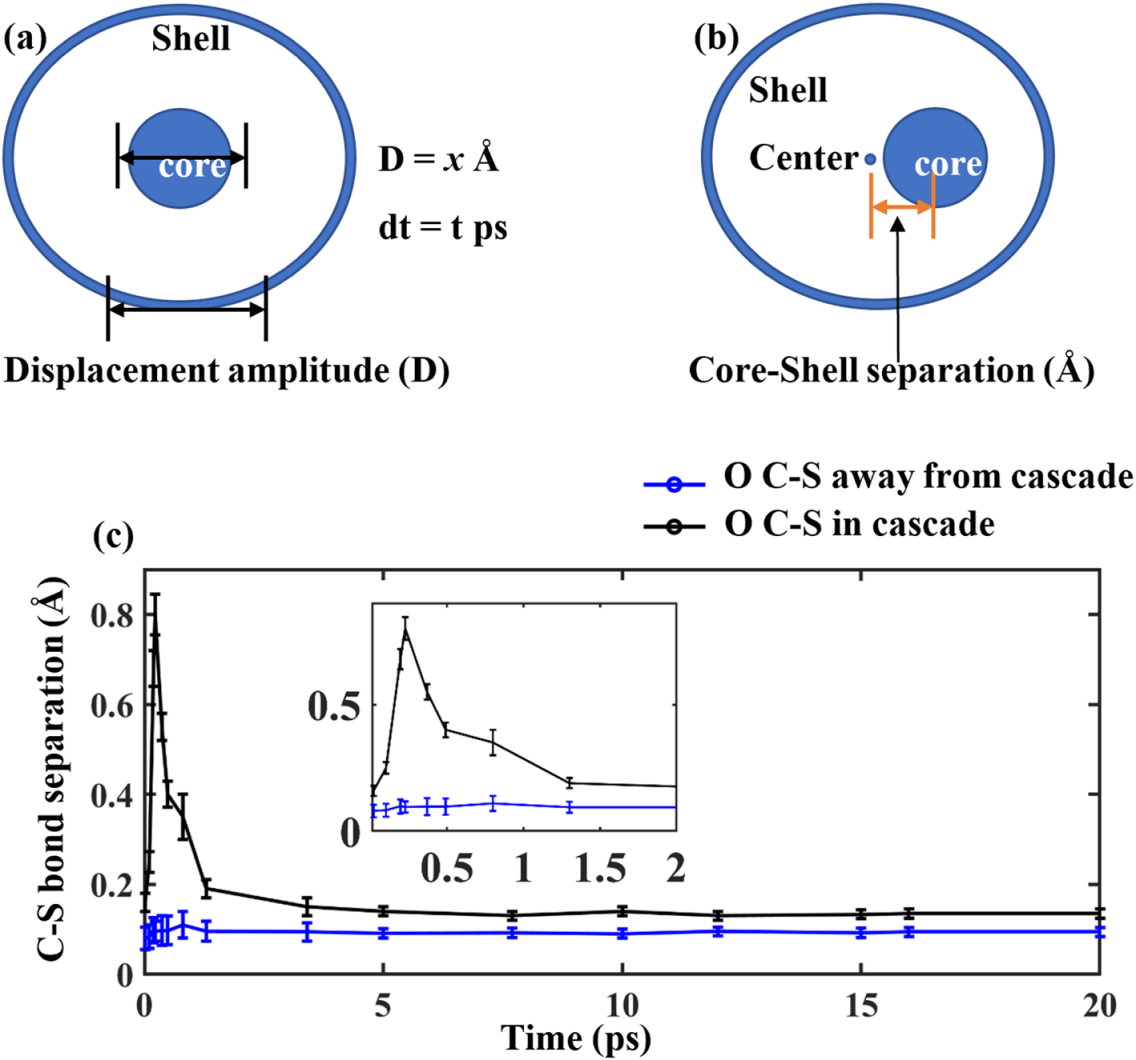
(**a**) Representation of a core–shell model for O atom showing the relative directions of motion. (**b**) Center to center separation distance created due to relative motion of a core and a shell. (**c**) Average separation distance between the cores and shells of O atoms in the cascade region (black) and in the non-cascade region (blue). Inset in Fig. 3c shows a magnification of the 0–2 ps time frames.

The maximum displacement of each particle was limited by using a variable timestep in LAMMPS, where the timestep size was adjusted such that the displacement of any particle did not exceed a maximum of 0.003 nm in any MD step. This constraint helped by keeping the core–shell separation distance limited in every timestep. However, the core–shell separation distance in the cascade region far exceeded that in the non-cascade region due to the high energy experienced during the collision events in the cascade region. This was verified by evaluating the relative distance between the core and the shell for select O atoms confined in the cascade region, and for select O atoms in the non-cascade region. Figure 4a explains the possible relative motion of the core and the shell and Fig. 4b depicts the core shell center-to-center separation distance. Around 4000 oxygen atoms in the cascade region (region surrounding the vacancy clusters in Fig. 3c) and around 60,000 oxygen atoms in the surrounding region (region away from the cluster in Fig. 3c) were selected for analysis, and their coordinates were used to calculate the average core–shell separation over 20 ps from the initiation of the cascade. Figure 4c shows that the O core–shell in the cascade region (black line) experienced up to 8 times higher displacement between 0 and 1.5 ps than in the non-cascade region (blue line), which is close to the magnitude mentioned for core–shell separation by Sahoo et al.^12^ for O in Li_2_TiO_3_. After 2 ps, the core–shell separation in the cascade region is only slightly higher (1.5–2 times) than than in the surrounding region.

The analysis of cascade simulation data reveals some interesting results about the kinetics involved in the background. As mentioned earlier, leveraging the variable timestep function allows the adjustment of timestep size to constrain the atomic displacements in a single timestep. The consequences of the variable timestep can be realized during the evolution of Frenkel pairs as an atomic displacement cascade propagates. The evolution of the cascade can be divided into supersonic, transonic, sonic and thermal annealing phases^37^.

In the supersonic and transonic phases, the LAMMPS code reduces the timestep size to a minimum to minimize the atomic displacements during the high K.E. period of PKA and thus prevent core–shell separation. When the Li PKA is launched, the velocity corresponding to the 15 keV case is around 6460 Å/ps (646,000 m/s). After roughly 86,000 MD steps, the KE drops to 0.1 eV where the corresponding velocity of the PKA is roughly 16 Å/ps (1600 m/s).

**Figure 5:**
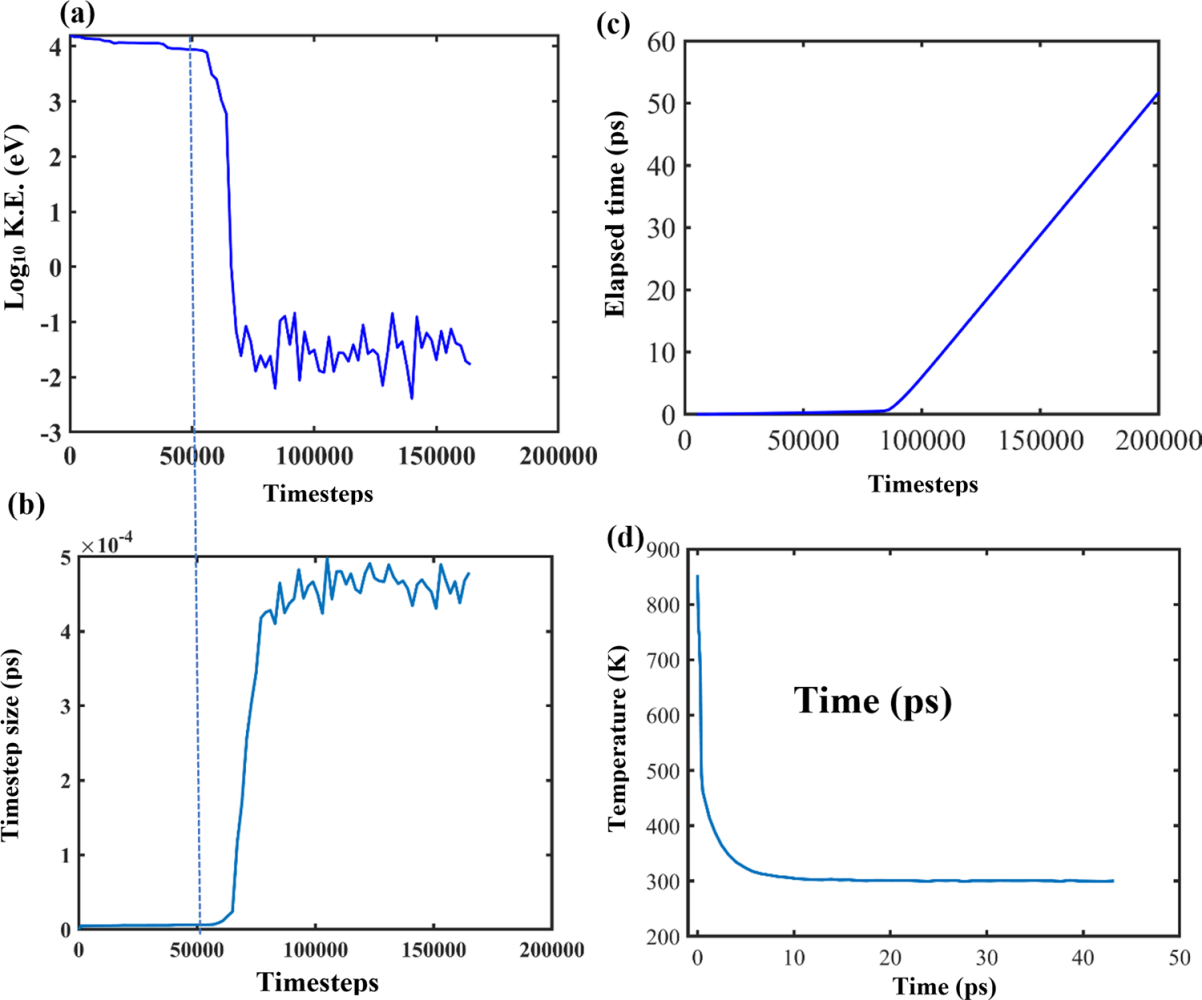
Evolution of system parameters with time/timestep elapsed, (**a**) K.E. variation with timesteps shown by a base-10 logarithmic scale, (**b**) timestep size variation with number of timesteps elapsed, the vertical dashed line marks the point of drop in PKA energy below 1 keV that allows the time step size to start increasing (**c**) total time elapsed as a function of timesteps, (**d**) temperature variation with total time elapsed.

The first three phases (supersonic, transonic, sonic), although not specifically differentiated, lie on the left of the vertical dashed line in Fig. 5a and b. The vertical dashed line denotes the 50,000 timestep mark. Past the 50,000 timestep mark, the kinetic energy of the PKA rapidly falls from 15 keV to sufficiently below 1 keV such that even the fastest atom does not meet the maximum allowable distance of X_max_ = 0.003 nm per timestep. As the PKA energy drops and the velocity reduces, LAMMPS allows larger timestep sizes in each iteration because now the energy of the atoms is lower and allowing a larger timestep does not pose a risk of high displacement of the O cores and shells. This is clearly shown by Fig. 5b where the timestep size increased rapidly after around 80,000 timesteps to the right of the dashed line. Thus, once the PKA energy has fallen to such a low magnitude, the LAMMPS code starts to increase the timestep value such that the condition of X_max_ = 0.003 nm of displacement per timestep is met by the fastest moving atom in the box. Therefore, we see almost a synchronous rise in timestep value with a fall in KE value.

Figure 5c represents the total time elapsed after the initiation of the PKA and complements the preceding figure with a sudden sharp increase after the decrease in PKA energy at around 80,000 timesteps. Correspondingly, the variation of temperature of the system with the elapsed time is shown in Fig. 5d. The maximum temperature was attained at the instance of the PKA initiation and dissipated rapidly with time. The thermostat plays a crucial role in limiting the maximum temperature of the system to around 850 K which is well below the melting temperature of these ceramics and hence there was no risk of phase transformation. Phase transformation of the γ phase may occur at 3.5 GPa and 1123 K^45,46^ resulting in the formation of α phase, but below 1093 K only the γ phase is stable. However, the K.E. of the cascade atoms will not be significantly affected by the change in the box size which is why the defect dynamics will remain largely unchanged.

**Figure 6:**
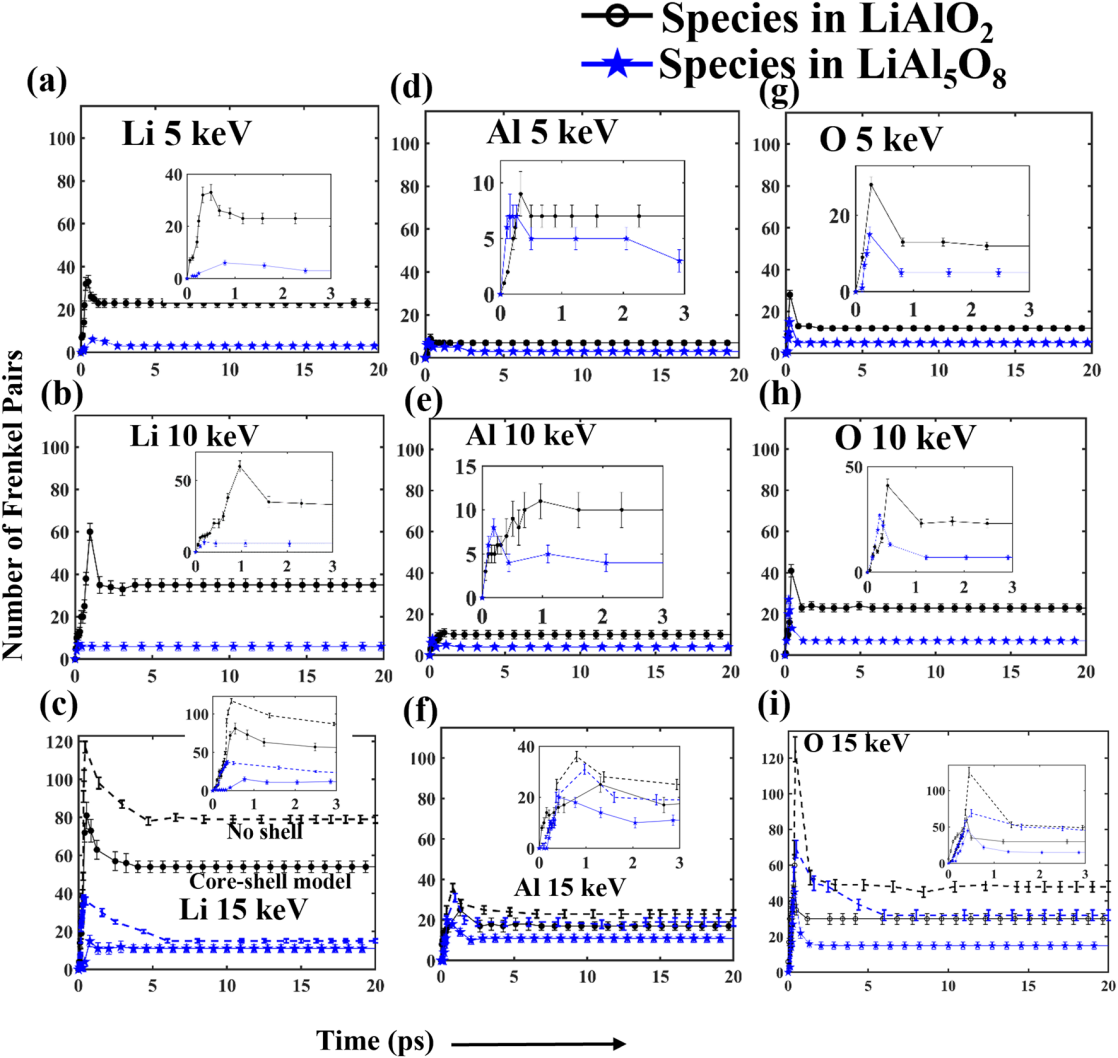
Evolution of Frenkel pairs for the individual species, (**a–c**) Li at 5, 10 & 15 keV, (**d–f**) Al at 5, 10 & 15 keV, and (**g**–**i**) O at 5, 10 & 15 keV. The black line with circled markers denotes the species in LiAlO_2_ and the blue line with pentagrams denotes the species in LiAl_5_O_8_. The inset in each figure shows a magnification of the 0–3 picoseconds time frame. The dashed lines in figure (**c**), (**f**) and (**i**) show the evolution of Frenkel pairs for the individual species when modeled without using a core–shell model for oxygen.

Figure 6 shows the variation of the number of Frenkel pairs with respect to the elapsed time during the cascade evolution for each individual species in the system. In the supersonic phase, the PKA penetrated through the lattice and displaced other atoms from their sites causing a sequence of high energy collisions. These collisions produced tertiary knock-on atoms that resulted in a collision cascade^47^. The supersonic phase typically lasted 0.1–0.2 ps in our simulation similar to previous work in metallic systems^35^, when the number of Frenkel pairs rose sharply in the system. After around 0.7 ps the transonic phase occurred where the primary knock-on initiated secondary and tertiary knock-on atoms, thereby triggering a cascade and causing an avalanche of Frenkel pairs as seen in Fig. 6 for all species. There was no identifiable distinction between the transonic and sonic phases from the Frenkel pair curves in the current systems, and they overlapped. The thermal annealing phase started after the peak of the Frenkel pairs had been reached. The atoms had exhausted the excess energy to create further defects and the recombination of interstitials with vacancies in the immediate vicinity became the dominant process. In the present system, this phase lasted roughly from around 2–20 ps, after which the number of Frenkel pairs became constant and stable.

The number of Frenkel pairs of individual species is plotted in Fig. 6a–i. All black lines with black circles denote the Frenkel pairs in LiAlO_2_ and the blue lines and pentagrams denote LiAl_5_O_8_. All species experienced at least 50% lower damage in LiAl_5_O_8_ than in LiAlO_2_. The number of Li Frenkel pairs in LiAlO_2_ after thermal annealing was 21, 37 and 56 as compared to 3, 5 and 17 in LiAl_5_O_8_, or ~ 3–5 times higher in LiAlO_2_. For Al and O at 5, 10 and 15 keV in Fig. 6d–f and g–i respectively, the number of Frenkel pairs in LiAlO_2_ was consistently 1.5–2 times higher than in LiAl_5_O_8_. From these observations, it is clear that in LiAlO_2_, the Li sublattice experienced the most damage, which was up to 60% of the total damage, consistent with the observations in ref.^17^. For LiAl_5_O_8_, the Li damage only accounted for about 25–30% of the total damage, with other sublattices experiencing about the same. It is worth noting that the Li atomic fraction is much smaller in LiAl_5_O_8_ relative to LiAlO_2_. The stable number of Li Frenkel pairs in LiAlO_2_ after thermal annealing increased from 21 at 5 keV to 57 at 15 keV as seen in Fig. 6a–c, which is a ~ threefold increase. The number of Al and O Frenkel pairs, however, increased only by a factor of 2 in LiAl_5_O_8_.

Displacements cascade simulations were also modeled in these two ceramics without the core shell model for oxygen. A comparison of the performance of the potential with and without the oxygen shells is shown in Fig. 6c, f and i for Li, Al and O respectively. Our investigation reveals that the cascade damage is overestimated in the absence of a core–shell model as shown in Fig. 6c, f and i. This is expected, because of energy losses associated with core–shell model that are absent in the cores only model. In the core–shell model, the core and the shell are essentially modeled as two concentric spheres connected by a spring. This spring loaded system makes the atomic collisions inelastic during a cascade, due to the conversion of kinetic energy into spring potential energy, thus moving the system away from elastic collisions. A simple energy conservation equation would look like

2 $$\frac{1}{2}{m}_{1}{u}_{1}^{2}+\frac{1}{2}{m}_{2}{u}_{2}^{2}+\frac{1}{2}k{x}_{initial}^{2}=\frac{1}{2}{m}_{1}{v}_{1}^{2}+\frac{1}{2}{m}_{2}{v}_{2}^{2}+\frac{1}{2}k{x}_{final}^{2}$$

where the kinetic energy loss term is represented by $\frac{1}{2}k{x}_{initial}^{2}-\frac{1}{2}k{x}_{final}^{2}$. This loss due to spring potential energy is absent in the cores only model where the collisions are more elastic in nature. Due to higher energy transferred during elastic collisions, the damage during the displacement cascades is overestimated and higher for all species Li (Fig. 6c), Al (Fig. 6f) and O (Fig. 6i).

**Figure 7:**
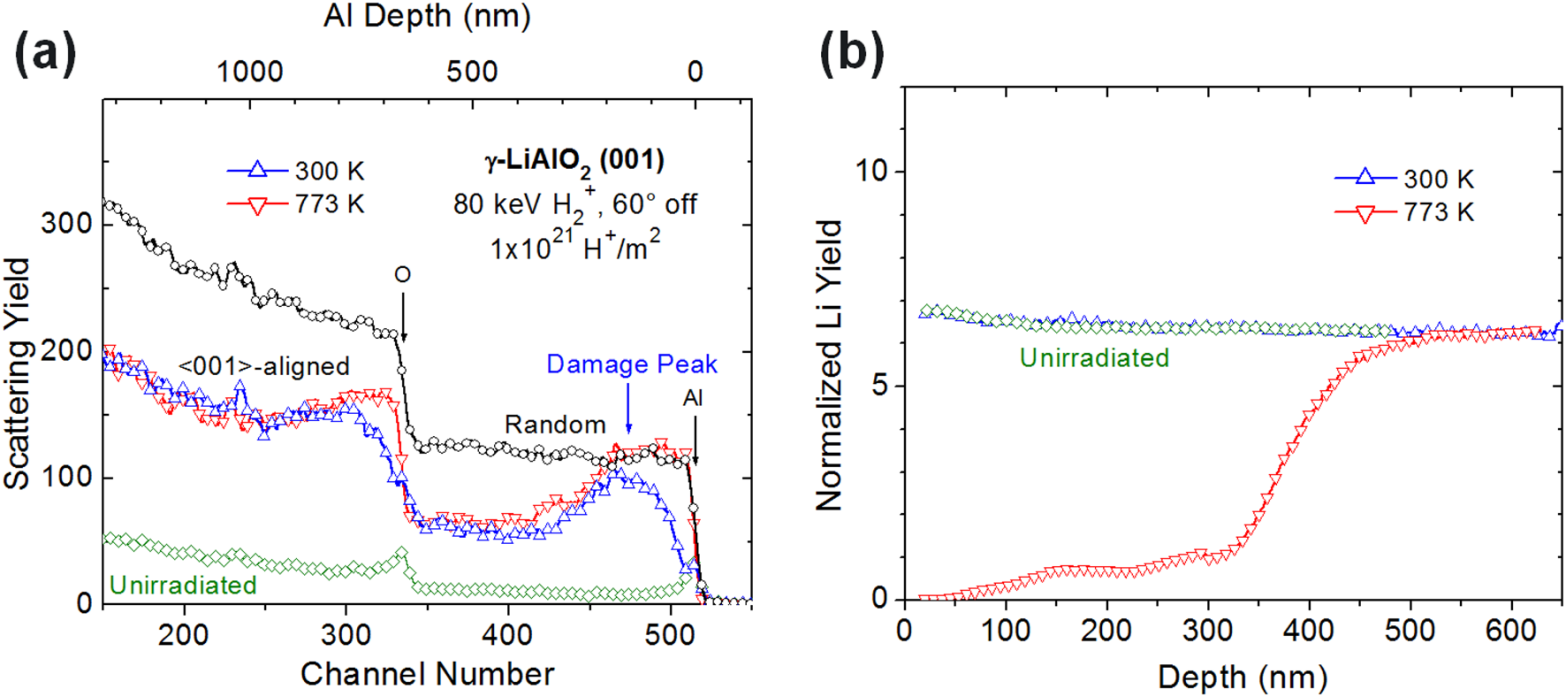
(**a**) 2 MeV He^+^ RBS/C spectra along the <001> axis in a γ-LiAlO_2_ single crystal irradiated 60° off normal with 80 keV H_2_^+^ molecular ions to 10^21^ H^+^/m^2^ at 300 and 773 K. Random and channeling spectra from an unirradiated area have also been included. (**b**) ToF–SIMS depth profiles of Li in the same samples^48^.

The dynamic process for the damage production in LiAlO_2_ is consistent with the experimental observations based on ion irradiation, followed by Rutherford backscattering spectrometry (RBS) along the <001>-axial channeling direction (RBS/C)^48^. The RBS/C spectra for a LiAlO_2_ single crystal irradiated to 10^21^ H^+^/m^2^ at 300 and 773 K are shown in Fig. 7a, along with the <001>-aligned spectra from an unirradiated area. A damage peak is clearly exhibited for the 300 K data as a result of the atomic collisions. In contrast, full amorphization in the irradiated depth region occurs at 773 K due to chemical decomposition^48^. From Fig. 7a, the relative disorder on the Al sublattice at the damage peak depth (~ 150 nm) is estimated to be 0.78 as viewed along the <001>-axial direction. The damage at 300 K is due to collision and not due to chemical decomposition as evinced in our simulations. To study the depth profile concentration of Li ions, time-of-flight secondary ion mass spectrometry (ToF–SIMS) was carried out in that work^48^. A ToF–SIMS plot of Li yield normalized to Al yield is shown in Fig. 7b. The results show that significant Li diffusion and release takes place near the surface region during ion irradiation at 773 K within 300 nm^48^ as compared to the unirradiated sample but no diffusion takes place at 300 K. This confirms that the Li damage noticed in our simulations is due to collision exclusively and not due to diffusion. These experimental data validate the simulations in this study, where the Li sublattice damage is predicted to be more than ~ 4 times the Al sublattice damage for all PKA energies.

**Figure 8:**
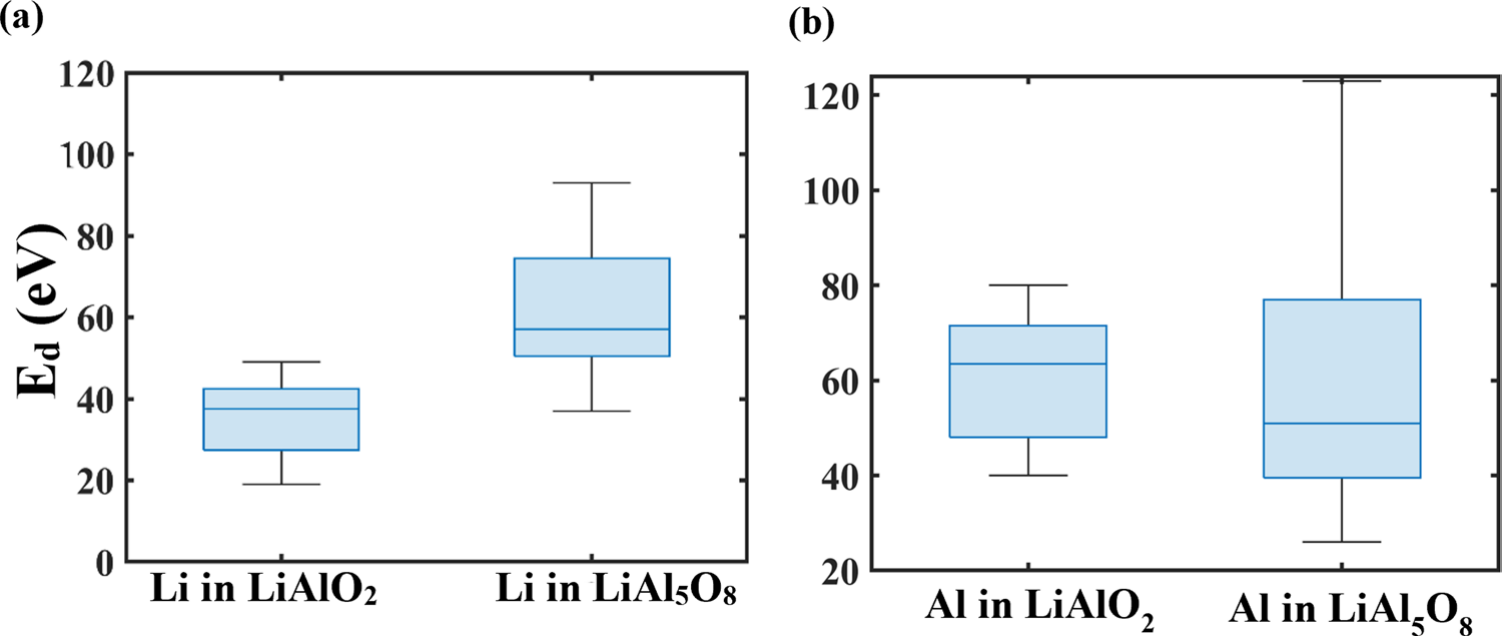
Li and Al displacement energies (eV) in LiAlO_2_ and LiAl_5_O_8_.

To identify the causes of these observations, we support the results with displacement threshold energy calculations as reported in our prior work^9^. We carried out displacement energy calculations in the 8 directions identical to those in which the PKA for cascades were initiated. The displacement threshold energies are shown in Fig. 8.

Figure 8 shows the E_d_ for Li and Al in 8 different crystallographic directions. Clearly, the E_d_ for Li atoms in LiAlO_2_ (median value = 37.5 eV) was lower than in LiAl_5_O_8_ (median value = 57 eV) in most directions, which is the primary reason that the Li sublattice undergoes much higher damage in LiAlO_2_. Al, on the other hand, had a higher median value in LiAlO_2_ (63.5 eV) than in LiAl_5_O_8_ (51 eV), but had a higher spread in E_d_ values in LiAl_5_O_8_. The number of directions sampled can be increased to get a more accurate comparison, but since the goal is to compare the E_d_ values in the same directions as the cascade PKA, we limited the sample size to these selected directions. The differences in E_d_ between the two ceramics are likely to be related to differences in crystal structure and cohesive energies. As explained in our previous work^9^, the potential allows a strongly ordered structure of Li–O bonds in LiAl_5_O_8_ as seen by sharp second, third and fourth neighbor peaks in the pair correlation functions. The Li–O peaks at longer distances are not as well defined in LiAlO_2_, which evinces that it may cost lower energy for cations to break the Li–O bond in LiAlO_2_ than in LiAl_5_O_8_.

**Figure 9:**
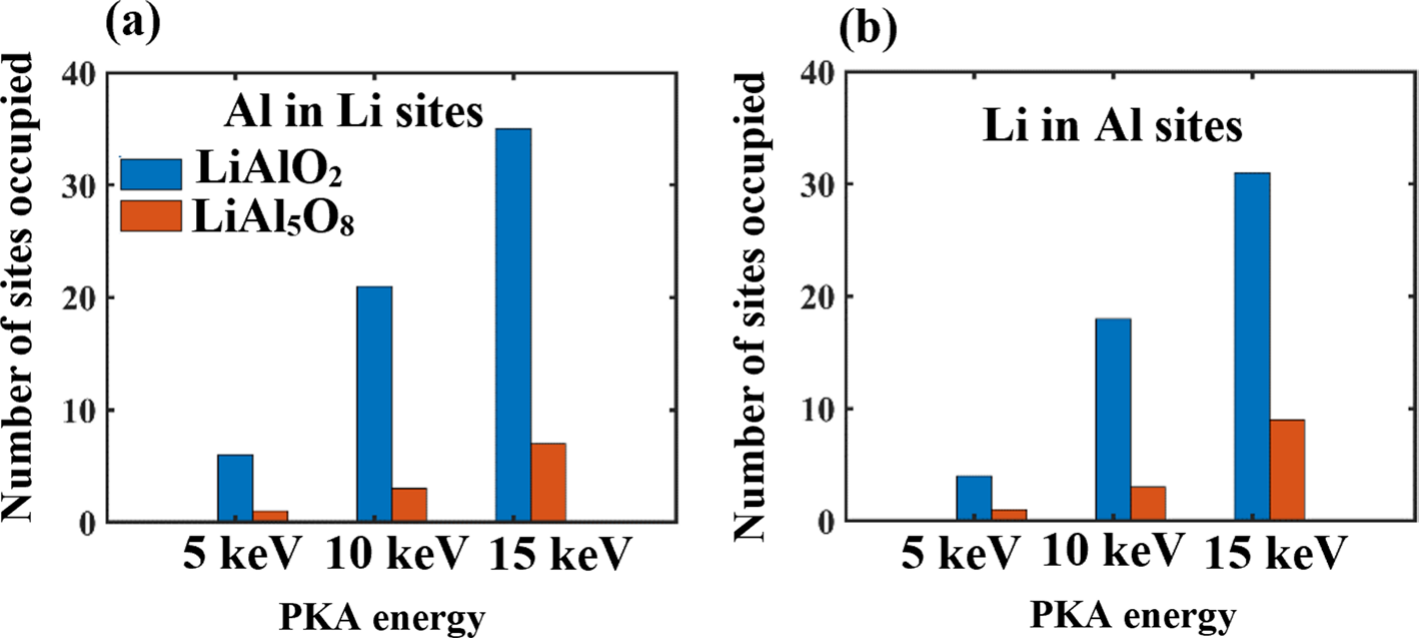
Number of sites occupied due to cation exchange as a function of PKA energy in LiAlO_2_ and LiAl_5_O_8_ with (**a**) showing the Al ions occupying the Li sites and (**b**) showing the Li ions occupying the Al sites.

Cation exchange analysis was performed to quantify the Li-Al cation antisite defects in both systems in Fig. 9. It is seen that there is a higher degree of cation exchange in LiAlO_2_ than in LiAl_5_O_8_ for both cases, i.e. Al ions in Li sites (Fig. 9a) and Li ions in Al sites (Fig. 9b). The reason for a higher cation exchange in LiAlO_2_ is the lower antisite defect formation energy of 1.76 eV as compared to that in LiAl_5_O_8_ with a value of 3.22 eV as calculated in our previous work^9^. This result suggests that it is more favorable for antisite defects to form in the LiAlO_2_ than in LiAl_5_O_8_ as seen in Fig. 9.

The present results show that fewer defects are produced in the primary damage state in LiAl_5_O_8_ than in LiAlO_2_ under identical nuclear stopping conditions. Cation chemical order is more likely to be retained in LiAl_5_O_8_ than in LiAlO_2_ under irradiation. LiAl_5_O_8_ is more likely to maintain structural and chemical integrity compared to LiAlO_2_ under irradiation resulting in superior radiation tolerance of LiAl_5_O_8_.

## Conclusion

A comparative study of the radiation resistance in LiAlO_2_ and LiAl_5_O_8_ using molecular dynamics was performed. Collision cascades were modeled by initiating PKAs with energies between 5 and 15 keV and the defect evolution was quantified in terms of Frenkel pairs generated during the cascade. The number of Li Frenkel pairs in LiAlO_2_ was 3–5 times higher than in LiAl_5_O_8_, while the number of Frenkel pairs for Al and O in LiAlO_2_ are ~ 2 times higher than in LiAl_5_O_8_. The primary cause for these observations appears to be high displacement threshold energies in LiAl_5_O_8_ for Li cations. The results highlight differences in defect production during radiation damage in the two ceramics that may hold the key to understanding Li burnup limits on structural integrity.

## Data Availability

The data and methods reported in this paper are available from the corresponding author upon reasonable request.
